# Rethinking the network determinants of motor disability in Parkinson’s disease

**DOI:** 10.3389/fnsyn.2023.1186484

**Published:** 2023-06-28

**Authors:** Dalton James Surmeier, Shenyu Zhai, Qiaoling Cui, DeNard V. Simmons

**Affiliations:** Department of Neuroscience, Feinberg School of Medicine, Northwestern University, Chicago, IL, United States

**Keywords:** dopamine, basal ganglia, circuit, Parkinson’s disease, plasticity, neuromodulation

## Abstract

For roughly the last 30 years, the notion that striatal dopamine (DA) depletion was the critical determinant of network pathophysiology underlying the motor symptoms of Parkinson’s disease (PD) has dominated the field. While the basal ganglia circuit model underpinning this hypothesis has been of great heuristic value, the hypothesis itself has never been directly tested. Moreover, studies in the last couple of decades have made it clear that the network model underlying this hypothesis fails to incorporate key features of the basal ganglia, including the fact that DA acts throughout the basal ganglia, not just in the striatum. Underscoring this point, recent work using a progressive mouse model of PD has shown that striatal DA depletion alone is not sufficient to induce parkinsonism and that restoration of extra-striatal DA signaling attenuates parkinsonian motor deficits once they appear. Given the broad array of discoveries in the field, it is time for a new model of the network determinants of motor disability in PD.

## Classical model of network dysfunction in PD

In the late 1980s, neuroscientists and clinicians embraced a new model of how the circuitry of the basal ganglia worked and how it went awry in Parkinson’s disease (PD) (Albin et al., 1989; DeLong, 1990; Gerfen, 1992). A key feature of the model was that there were two opposing projection systems in the striatum. The so-called “direct” pathway was anchored by striatal GABAergic spiny projection neurons (SPNs) that innervated substantia nigra pars reticulata (SNr) and globus pallidus interna (GPi) GABAergic neurons at the interface of the basal ganglia with the rest of the brain. The “indirect” pathway was anchored by a distinct group of striatal GABAergic SPNs that innervated the globus pallidus externa (GPe); the GPe GABAergic neurons in turn innervated the same interface nuclei and a neighboring glutamatergic nucleus, the subthalamic nucleus (STN). In this circuit, activation of direct pathway SPNs (dSPNs) by cortical pyramidal neurons led to inhibition of basal ganglia output through SNr and GPi, enhancing the excitability of thalamic motor nuclei and promoting movement. In contrast, activation of indirect pathway SPNs (iSPNs) suppressed GPe activity, enhancing basal ganglia GABAergic outflow both by disinhibiting the interface nuclei directly and by enhancing excitatory STN input to them – thus, suppressing movement.

This opponent process system was modulated by striatal dopamine (DA) release from axons of substantia nigra pars compacta (SNc) neurons. Work around this time suggested that DA stimulation of D1 DA receptors (D1Rs) expressed by dSPNs enhanced their excitability and responsiveness to cortical input, whereas DA stimulation of D2 DA receptors (D2Rs) expressed by iSPNs did the opposite (Gerfen and Surmeier, 2011). Through this modulatory network, bursts of action potentials in SNc DAergic neurons and transient elevations in striatal DA release would promote activation of dSPNs and suppress activation of iSPNs, resulting in movement. Conversely, inhibition of SNc DAergic neuron activity and a transient reduction in striatal DA release would do the opposite and suppress movement. Thus, in PD, the loss of striatal DA release biased the striatal circuitry toward the indirect pathway and the suppression of movement (Albin et al., 1989; DeLong, 1990).

This simple model has been of enormous heuristic value. It created a framework for exploration and discovery. In recent years, technical advances have accelerated the pace of basal ganglia research and have increased our ability to rigorously test key features of the model. For example, the development of bacterial artificial chromosome (BAC) transgenic mice in which dSPNs or iSPNs expressed fluorescent proteins or Cre recombinase, the emergence of optogenetics and chemogenetics, and the ability to monitor the activity of genetically defined neurons in awake, behaving animals have allowed key features of the model to be put to the test. This growing body of literature has made it clear that the now “classical” model is in need of significant revision.

## Problems with the classical model

There have been a number of excellent reviews detailing how work in the last several decades has confirmed some aspects of the classical model, but also how these discoveries have revealed its shortcomings (Levy et al., 1997; Nambu, 2008; Obeso and Lanciego, 2011; Quiroga-Varela et al., 2013; Wichmann, 2019; Zhai et al., 2019). Among these shortcomings are (1) the failure to consider the complexity of the basal ganglia circuitry and that iSPNs and dSPNs do not simply act as opposing agents in movement control, (2) the failure to consider the role of SPN ensembles or networks (as opposed to individual neurons) in coding movement, (3) the failure to consider the intrastriatal circuitry, (4) the failure to consider that DA not only modulates the moment-to-moment excitability of neurons, but also long-term plasticity, (5) the failure to consider alternative signaling linkages of the G protein-coupled receptors (GPCRs) mediating the effects of DA (Fuxe et al., 2010; Gomes et al., 2016), (6) the failure to consider that DA modulates essentially all of the basal ganglia, not just the striatum, (7) the failure to consider that DA depletion triggers a broad array of alterations in the basal ganglia circuitry, and (8) the failure to consider the role of rhythmicity and synchrony of spiking – rather than just mean rate in the emergence of movement deficits.

Reviewing all these issues is beyond the scope of this short review. Rather, the focus here will be on two topics raised by our recent study of a progressive model of PD (González-Rodríguez et al., 2021). The first topic will be how DA depletion alters the striatal circuitry. The second topic will be how the release of DA outside the striatum, specifically dendritic release in the mesencephalon, might regulate the ability of the basal ganglia to influence motor circuits.

## The striatal circuitry – beyond a simple opponent process

In the original model proposed by Albin et al., iSPNs and dSPNs were envisioned to work in opposition to one another to either initiate or suppress movement. A watershed discovery in the field was the recognition that in fact iSPNs and dSPNs work together to control purposeful movement and are co-activated at movement initiation (Cui et al., 2013; Jin et al., 2014; Tecuapetla et al., 2016). How this coordinated activity is orchestrated, its dependence upon DA and its relationship to movement control remain to be resolved – but it is now widely accepted that iSPNs and dSPNs do not simply work in opposition to one another (Sheng et al., 2019; Lopez-Huerta et al., 2021). From the standpoint of the classical model, a particularly problematic observation is that movement initiation is accompanied by a transient elevation in striatal DA release (Howe and Dombeck, 2016; Howard et al., 2017). In the classical model, this elevation in DA release should result in the activation of dSPNs and suppression of iSPNs, not co-activation. Clearly, there are other factors in play.

One of these other factors is the network of striatal interneurons that regulate the activity of SPNs. Recent studies have started to link specific types of striatal interneurons to different aspects of motor function and learning (Bradfield et al., 2013; Burguiere et al., 2013; Owen et al., 2018; Holly et al., 2019; Kaminer et al., 2019). A key node in this intrastriatal circuitry is the “giant,” aspiny cholinergic interneuron (ChI). ChIs are autonomously active (like SNc DA neurons) (Bennett and Wilson, 1999); this autonomous activity is modulated by glutamatergic neurons in the thalamic parafascicular nucleus (PFN) and cortical regions, like the cingulate cortex, involved in monitoring internal state (Bradfield et al., 2013; Doig et al., 2014; Gonzales and Smith, 2015; Kosillo et al., 2016; Melendez-Zaidi et al., 2019; Tanimura et al., 2019). Consistent with this extrinsic connectivity, ChIs have been implicated in shifting action selection (state transitions) with changing environmental contingencies and sequencing movement (Bradfield et al., 2013; Matamales et al., 2016; Howe et al., 2019).

**FIGURE 1:**
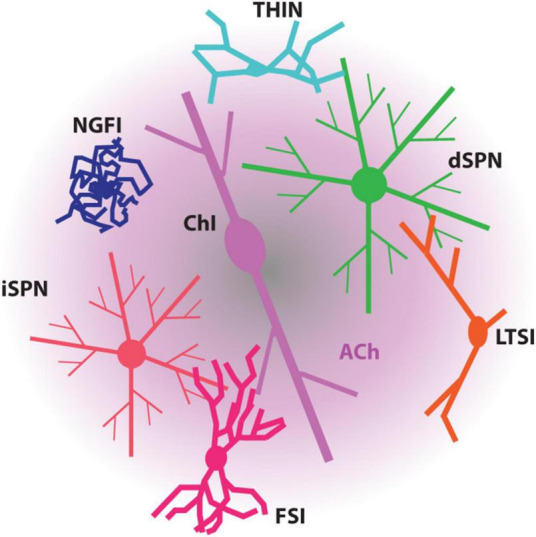
Diagram depicting the modulation of SPNs and striatal interneurons by ACh. ACh, mostly released by ChIs, influences all these striatal neurons through the muscarinic receptors and/or nicotinic receptors they express. FSI, fast-spiking interneuron; LTSI, low-threshold spiking interneuron; NGFI, neurogliaform interneuron; THIN, tyrosine hydroxylase-expressing interneuron.

**FIGURE 2:**
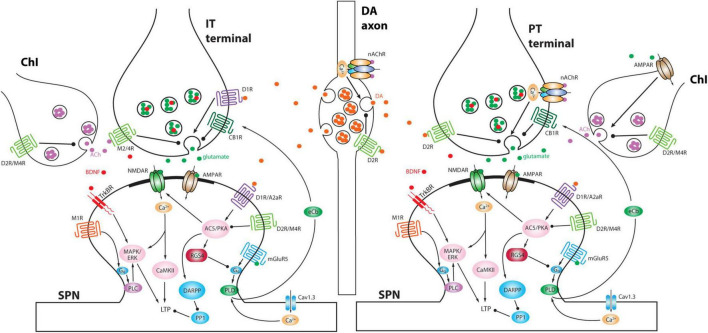
Schematic depicting differential modulation of presynaptic glutamate release at IT versus PT terminals and postsynaptic signaling pathways by DA and ACh. Moreover, DA axons and ChIs interact with each other. Note that ChI is preferentially activated by PT input compared to IT input and in turn ACh activates nAChRs on PT terminals to elicit a delayed second phase of excitation (Morgenstern et al., 2022). A2aR, adenosine receptor 2a; AC5, adenylyl cyclase 5; AMPAR, α-amino-3-hydroxy-5-methyl-4-isoxazolepropionic acid receptor; BDNF, brain-derived neurotrophic factor; CaMKII, Ca^2+^/calmodulin-dependent protein kinase II; CB1R, cannabinoid receptor type 1; D1R, dopamine D1 receptor; D2R, dopamine D2 receptor; DARPP, dopamine- and cAMP-regulated phosphoprotein of Mr 32,000; eCb, endocannabinoid; ERK, extracellular signal-regulated kinase; M1R, muscarinic ACh receptor 1; M2/4R, muscarinic ACh receptor 2 or 4; MAPK, mitogen-activated protein kinase; mGluR5, metabotropic glutamate receptor 5; NMDAR, N-methyl-D-aspartate receptor; PKA, protein kinase A; PLC, phospholipase C; PLD, phospholipase D; PP1, protein phosphatase 1; RGS4, regulator of G-protein signaling 4; TrkBR, tropomyosin related kinase B receptor.

The impact of ChIs on the striatal circuitry and SPNs is largely mediated by acetylcholine (ACh) release from highly arborized axons (but see Dautan et al., 2014; Figure 1). The dimensions of these axons are similar to those of mesostriatal dopaminergic neurons and may have several hundred thousand release sites (Zhou et al., 2002; Matsuda et al., 2009). Although spatially diffuse, ACh signaling is temporally constrained by robust expression of acetylcholinesterase (AChE) in the striatum (Graybiel and Ragsdale, 1978). This allows for both rapid ACh signaling through ionotropic nicotinic ACh receptors (nAChRs) and slow signaling through metabotropic muscarinic ACh receptors (mAChRs). Although our understanding of ChI connectivity is still emerging, a burst of ChI activity engages nAChRs to rapidly drive glutamate release from pyramidal tract (PT) terminals and to activate both tyrosine hydroxylase-positive GABAergic interneurons (THINs) and neurogliaform GABAergic interneurons (NGFIs) – all of which target SPN dendrites (Tepper et al., 2018; Kocaturk et al., 2022; Morgenstern et al., 2022; Figures 1, 2). In parallel, ACh inhibits glutamate release from M4 mAChR-expressing intratelencephalic (IT) glutamatergic terminals (Figure 2) and enhances the excitability of M1 mAChR-expressing iSPNs (Shen et al., 2005, 2007; Day et al., 2008; Crittenden et al., 2021; Pancani et al., 2023). These actions are consistent with a “disruptive,” set-shifting role of ChIs. Precisely how ChIs are modulating dSPNs is less clear-cut. In contrast to iSPNs, dSPNs express both M4 and M1 mAChRs (Yan et al., 2001). While dendritic M4 mAChRs clearly blunt the impact of D1R signaling that enhances the response to depolarizing glutamatergic input (Shen et al., 2015), whether they modulate excitability in the absence of DA signaling is unclear. Somewhat paradoxically, in the peri-somatic region, M4 mAChRs appear to enhance excitability (Hernandez-Flores et al., 2015). In contrast, M1 mAChRs enhance spike generation when dSPNs are near spike threshold possibly by targeting several ion channels (Perez-Rosello et al., 2005; Shen et al., 2005; Perez-Burgos et al., 2008). Sorting out these seemingly disparate effects on dSPN excitability remains a challenge.

Interestingly, ChI activity rises rapidly at the onset of spontaneous movements and movement transitions (Howe et al., 2019). In the dorsolateral striatum, this initial burst of activity is likely to be driven by activity in the PFN and by glutamate release from DA terminals (Schulz et al., 2009; Cai and Ford, 2018; Tanimura et al., 2019). Given that ACh significantly enhances the excitability of iSPNs, their co-activation with dSPNs during the onset of movement could be dependent upon ChI activity. In fact, transient elevation in striatal DA release at movement onset may be driven in part by ChIs and activation of presynaptic nAChRs on DA terminals (Figure 2; Zhou et al., 2001; Ding et al., 2010; Threlfell et al., 2012; Berke, 2018; Kramer et al., 2022; Liu et al., 2022). Subsequent work has challenged this idea though, showing there is a correlation between striatal DA release and the spiking of cell bodies in the mesencephalon (Azcorra et al., 2022).

Other players are likely to participate in the initial elevation in the probability of iSPN spiking. In addition to both cortical and thalamic excitatory input to iSPNs (Ding et al., 2010; Tanimura et al., 2016), extracellular adenosine levels in the striatum rise at the onset of spontaneous movements, leading to increased activation of A2aRs and protein kinase A (PKA) signaling in iSPNs (Ma et al., 2022). As PKA signaling should enhance postsynaptic excitability, this could contribute to the pattern of iSPN activity. That said, the kinetics and sub-cellular (dendritic) distribution of this modulatory pathway needs to be worked out. Nevertheless, it has also been found that iSPN D2Rs were only capable of inhibiting baseline A2aR signaling, but not that produced by the movement-related elevation in adenosine; this suggests that D2R coupling to adenylyl cyclase (AC) might be saturated by ambient DA levels in the striatum, making the transient elevation in DA levels associated with movement essentially irrelevant to DA modulation of iSPNs, contrary to the classical model.

In addition to ChIs, the striatum has a variety of other striatal GABAergic interneurons (defined by their synaptic connectome, physiology, protein co-expression and axonal fields) (Figure 1; Tepper et al., 2018). Essentially all of these interneurons express DA and ACh receptors, further complicating the model of how the timing and magnitude of SPN activity is controlled.

## Striatal adaptations in PD – the striatum is not static

A central tenet of the classical model is that the motor disability in PD is a consequence of an imbalance in the moment-to-moment excitability of iSPNs and dSPNs following striatal DA depletion (Albin et al., 1989). As noted above, deficits in striatal DA release are now known to induce a broad array of alterations in intrastriatal circuitry beyond those originally envisioned.

In the healthy striatum, DA has at least two major functions (Klaus et al., 2016; Berke, 2018; Coddington and Dudman, 2019; Markowitz et al., 2023). One is to facilitate the activity of neuronal ensembles associated with movement. This function is in alignment with the classical model. The other is to link action outcomes (either explicit or implicit) to the probability that the striatal ensembles associated with those actions will be engaged in the future. This function is critical to exploratory behaviors, reinforcement learning, goal-directed action, and habit. To this end, DA promotes lasting changes in striatal circuitry. The best characterized of these changes are in the strength of corticostriatal glutamatergic synapses formed on dendritic spines of SPNs (Gerfen and Surmeier, 2011; Lerner and Kreitzer, 2011). A transient elevation in DA release associated with explicit or implicit reward is thought to increase the strength of recently active glutamatergic synapses on dSPNs and to decrease the strength of those on iSPNs. In this way, the next time the associated cortical and thalamic circuits become active, it becomes more likely that dSPNs associated with the rewarded action become active, and less likely that iSPNs associated with that action are engaged – leading to striatal endorsement of the rewarded action. Conversely, when an action leads to a negative outcome, striatal DA levels transiently fall, decreasing the strength of recently active glutamatergic synapses on dSPNs and increasing the strength of those on iSPNs – leading to striatal vetoing of the un-rewarded or punished action.

Although there remain a broad range of unanswered questions about precisely how this is played out, this model of cell-specific, bidirectional regulation of synaptic strength by DA has considerable experimental support (Zhai et al., 2019). In dSPNs, increased D1R signaling promotes the induction of long-term potentiation (LTP) of corticostriatal glutamatergic synapses, whereas a drop in D1R signaling and concomitant elevation in M4 mAChR signaling promotes the induction of long-term depression (LTD) at nominally the same synapses (Figure 2; Shen et al., 2008, 2015; Yagishita et al., 2014). In iSPNs, a transient drop in D2R signaling and concomitant elevation in A2aR signaling promotes the induction of LTP at corticostriatal synapses, whereas an elevation in D2R signaling promotes the induction of LTD (Figure 2; Shen et al., 2008, 2015; Iino et al., 2020). In both cell types, there are other determinants of the induction and expression of long-term synaptic plasticity, but the basic framework aligns with the reinforcement learning model and the role of DA as a “teacher”. Complementing these homosynaptic forms of plasticity, DA also influences a heterosynaptic form of LTD at glutamatergic synapses on SPNs that is induced by nitric oxide (NO) release by a subtype of striatal interneurons (Park and West, 2009; Rafalovich et al., 2015). The key point here is that striatal DA, as a learning signal, allows action outcomes to shape future motor behavior through DA-dependent forms of synaptic plasticity in SPNs.

In the parkinsonian state, the linkage between action outcome and striatal DA release is broken. The striatal circuitry very likely “interprets” the persistent deficit in DA release as a negative outcome for any and all actions. This has several consequences for striatal plasticity. One is that there should be a standing bias toward strengthening of corticostriatal synapses on iSPNs and weakening of those on dSPNs. The experimental data is largely consistent with this conjecture. Following near-complete lesions of the striatal DA innervation, there is a significant increase in the number of large, “perforated” axospinous synapses on iSPNs and a shift in the distribution of unitary synaptic strengths toward larger values (Peterson et al., 2012; Zhang et al., 2013; Fieblinger et al., 2014). This is what would be predicted from a drop in D2R signaling, which disinhibits A2aR coupling to AC, and a rise in M1 mAChR signaling (resulting from disinhibition of ChIs).

But there is an interesting twist to this story. In parallel with the appearance of potentiated synapses, the total number of iSPN axospinous synapses of both cortical and thalamic origin falls by roughly a third (McNeill et al., 1988; Stephens et al., 2005; Zaja-Milatovic et al., 2005; Day et al., 2006; Schuster et al., 2009; Villalba et al., 2009; Nishijima et al., 2014; Suarez et al., 2014; Gagnon et al., 2017; Shen et al., 2022; Tanimura et al., 2022), leaving total synaptic strength constant (Fieblinger et al., 2014). There are two potential explanations for this synaptic remodeling. One is that the loss of inhibitory D2R signaling, elevation in M1 mAChR signaling and potentiation of glutamatergic synapses push the average spiking rate of iSPNs above a “set-point” and engage homeostatic mechanisms aimed at normalizing spiking rate. In other cell types it is clear that sustained deviations in spiking rate from a certain set-point are capable of inducing alterations in intrinsic excitability and synaptic strength that normalize activity (Marder and Goaillard, 2006; Turrigiano, 2012). In models of late-stage PD, the intrinsic excitability of iSPNs, as judged by spiking in response to intra-somatic current injection, falls in parallel with the pruning of dendritic synapses, consistent with the attribution of these adaptations to a homeostatic mechanism (Fieblinger et al., 2014). However, in the prodromal stage of the MCI-Park model of PD where there is a loss of striatal DA release in advance of parkinsonian motor disability, there is pruning of dendritic synapses without a clear change in somatic intrinsic excitability – arguing that somatic spiking has not changed significantly (Zhai et al., unpublished observations). One possible explanation for this dichotomy is that in the prodromal state, local dendritic mechanisms are capable of compensating for the regional loss of DA signaling, but as basal ganglia pathophysiology begins to disrupt extrinsic activity (in the cerebral cortex, thalamus, and brainstem), the synaptic drive on iSPNs overwhelms the dendritic adaptations leading to the engagement of intrinsic homeostatic plasticity in the somatic region.

It is also possible that the iSPN synaptic pruning is driven by aberrant plasticity mechanisms and is not homeostatic. In most models, with the loss of DA signaling, ChIs become more responsive to thalamic glutamatergic input and ACh release is disinhibited (MacKenzie et al., 1989; DeBoer et al., 1996; Ding et al., 2006; Sanchez et al., 2011; Chuhma et al., 2014; Straub et al., 2014; Tanimura et al., 2019; Padilla-Orozco et al., 2022; c.f., McKinley et al., 2019; Choi et al., 2020). These adaptations are consistent with a disruptive, set-shifting role for ChIs as striatal DA levels fall (described above). Elevated ACh signaling will not only enhance the postsynaptic mechanisms underlying the induction of LTP in iSPNs (Crittenden et al., 2021), but it will have presynaptic effects (Figure 2). Both iSPNs and dSPNs are innervated by two classes of corticostriatal axons: those arising from ipsilateral and contralateral IT neurons and those from ipsilateral PT neurons (Ballion et al., 2008; Kress et al., 2013; Shepherd, 2013; Deng et al., 2015). IT presynaptic terminals are invested with M2/4 mAChRs (and D1Rs), whereas PT terminals have nAChRs (and D2Rs) (Figure 2; Morgenstern et al., 2022; Pancani et al., 2023). Thus, with a drop in striatal DA and an elevation in ACh, IT terminals should be persistently inhibited whereas PT terminals should be persistently facilitated. As pre- and post-synaptic functions are often scaled in a coordinated fashion (Murthy et al., 2001), a persistent presynaptic depression of glutamate release by IT terminals is likely to be followed by a down-sizing of spine size that could ultimately result in frank synapse elimination. If this were the case, then iSPNs in the PD brain should become dominated by ipsilateral PT cortical input, disrupting the normally distributed interaction between IT and PT pathways in controlling striatal circuitry (Park et al., 2022). This conjecture is consistent with contralateral sensory neglect observed in unilateral PD models (Ketzef et al., 2017), as well as the more robust response to ipsilateral motor cortex stimulation (Mallet et al., 2006).

The experimental evidence in support of aberrant weakening of corticostriatal synapses on dSPNs is less compelling, but largely consistent. The initial descriptions of striatal spine and synapse reorganization following lesioning of DA neurons found little evidence of significant changes in dSPNs, despite later evidence that the somatic excitability of dSPNs rose in a homeostatic manner (Day et al., 2006; Fieblinger et al., 2014). Subsequent functional analyses found a significant reduction in the strength of axospinous glutamatergic synapses on dSPNs following DA depletion (Fieblinger et al., 2014), consistent with the notion that elevated M4 mAChR and loss of D1R signaling biased dSPNs toward LTD. About this time, work from other groups using intrastriatal 6-OHDA lesioning approaches (rather than medial forebrain bundle injection) claimed that there was a loss of axospinous synapses on both iSPNs and dSPNs (Suarez et al., 2014, 2016). This assertion aligned with work in primates following MPTP lesioning of DA neurons where there was spine loss in both SPN cell types (Villalba and Smith, 2013). The reasons for the apparent discrepancy appear to be largely methodological. One issue was when the connectivity assay was performed after lesioning. In the primate work, the assays were run months after the initial insult, whereas in mice they were done about a month afterward. If the post-lesion survival time in rodents was extended to 2 months, then there was a clear reduction in dSPN spine density (Graves and Surmeier, 2019). How then to explain the rodent work claiming early spine loss in dSPNs? Conventional optical methods for visualizing SPN spines can significantly underestimate the spine density estimates because so many SPN spines are long and thin (Wilson et al., 1983; Wilson, 1994). In our hands, by rendering z-stacks derived from two-photon laser scanning microscopy of SPNs in *ex vivo* brain slices, spine density estimates typically are around 1–1.5 spines/μm (Fieblinger et al., 2014; Graves and Surmeier, 2019), slightly below those estimated by Wilson et al., suggesting that some thin spines were being missed. With the attenuation of synaptic strength at axospinous synapses on dSPNs (Fieblinger et al., 2014), making this error becomes even more likely, potentially accounting for some of the discrepancy (Alberquilla et al., 2020). Another key difference between the rodent studies was the site of 6-OHDA injection. In those studies reporting early dSPN spine loss, 6-OHDA was administered intrastriatally. This approach results in robust striatal inflammation (Pabon et al., 2011), which may drive spine loss through microglial activation (Spangenberg et al., 2016).

The take-home point is that the synaptic architecture of SPNs which is critical to ensemble organization and the chaining together of meaningful movement syllables is disrupted in PD. In the healthy brain, this synaptic architecture is continuously tuned or taught by time-varying DAergic signals related to both explicit and implicit reinforcement. This teaching enables appropriate goal-directed actions, spontaneous exploration and habit. With the lesioning of DA neurons, the striatal teacher goes on strike, leading to synaptic changes that are unrelated to outcomes or events in the world. Although levodopa may alleviate the motor disability in PD in part by “re-balancing” the excitability of iSPNs and dSPNs, it does not restore the normal synaptic architecture of SPNs, it does not restore the temporal pattern of DA release, and it does not normalize SPN ensembles during behavior (Parker et al., 2018; Serrano-Reyes et al., 2022). Rather, as outlined below, the ability of levodopa to alleviate motor deficits in PD may be largely a consequence of suppressing pathological activity patterns in the basal ganglia output nuclei, allowing other brain regions to take over motor control.

## Beyond the striatum – the contribution of extra-striatal DA signaling to PD motor symptoms

In the last few decades, it has become apparent that the basal ganglia circuitry downstream of the striatum is more complex than originally thought. Moreover, each of the nuclei processing striatal output – the GPe, the GPi (entopeduncular nucleus in rodents), and the SNr is directly modulated by local DA release. This is also true for the STN, which links GPe, GPi, and SNr (Rommelfanger and Wichmann, 2010). Although there are many unanswered questions about how DA is acting in this part of the brain, the simple view at this point is that it is acting to enhance the coupling of dSPNs to the GPi/SNr and to diminish that of iSPNs through the indirect pathway. The uncoupling of the indirect pathway appears to be accomplished largely through presynaptic D2-class receptors that inhibit transmitter release (Hadipour-Niktarash et al., 2012). Thus, as in the striatum, DA release in the rest of the basal ganglia seems to be promoting movement.

But the functional importance of DA in this part of the basal ganglia has been easy to dismiss and difficult to rigorously assess. It has been easy to dismiss because the usual markers of DA signaling (e.g., tyrosine hydroxylase immunoreactivity) in these regions is much lower than that in the striatum, where the terminal arbors of DA axons reside. However, what is commonly ignored is that while there may be many fewer DA release sites in this part of the basal ganglia, there are far fewer DA uptake sites (i.e., DA transporter or DAT) (Rice et al., 2011). This allows DA released from en passant synapses to diffuse farther and act for a longer duration, much like a paracrine hormone – in contrast to the striatum, where the actions of released DA are rapidly terminated by DAT uptake of extracellular DA (Rice et al., 2011).

Losing the DA modulation of synaptic coupling in the extra-striatal basal ganglia is very likely to be an important factor in the emergence of synchronous, oscillatory phenomena in PD (Bergman et al., 1998; Raz et al., 2000; Bevan et al., 2002; Levy et al., 2002). Neurons in each one of the nuclei downstream of the striatum spike autonomously at relatively high rates. This autonomous spiking allows for rapid, bidirectional regulation of signals conveyed to motor circuits in the thalamus, mesencephalon and brainstem. But it also creates a network mechanism for synchronous oscillation given the robust synaptic coupling between the GPe – a GABAergic nucleus – and the STN – a glutamatergic nucleus. On the face of it, the reciprocal connectivity between an excitatory and inhibitory nucleus should create a Wilson-Cowan oscillator (Wilson and Cowan, 1972). This does not happen in healthy animals, presumably because DA dampens the strength of the connections between the nuclei and allows external synaptic input to drive transient changes in spiking rate and disrupt oscillation. In PD, when DA levels fall, the natural tendency for this part of the circuit to oscillate appears to be unleashed. Indeed, sustained, synchronous oscillatory activity in this part of the basal ganglia circuitry is a hallmark of PD motor deficits (Hammond et al., 2007).

Another region where DA signaling is undoubtedly crucial to normal basal ganglia function is the SNr. In the late 1970s, it was discovered that SNc DA neurons release DA from their dendrites (Geffen et al., 1976; Korf et al., 1976; Cheramy et al., 1981). Ventral tier DA neurons sitting at the border of SNr send their dendrites into the neuropil of the SNr, often forming beautiful “bouquets” (Crittenden et al., 2016). Although a number of studies have shown that somatodendritic DA signaling has a significant impact on SNr circuitry (Rice and Patel, 2015; Caceres-Chavez et al., 2018) and plays a key role in the ability of levodopa-derived DA to alleviate PD symptoms (Robertson and Robertson, 1988; Robertson, 1992), the focus of the field has remained on the striatum. There are many reasons for this turn of events, including a number of studies that are nominally consistent with the classical model (Kravitz et al., 2010; Alcacer et al., 2017). Aside from the experimental advantages of the striatum (it is large and accessible), it has been difficult to isolate the contributions of extra-striatal DA release to behavior and PD motor deficits in animal models. Toxin models, like the 6-OHDA model, which have dominated the field for decades, induce rapid axonal and somatodendritic degeneration of DA neurons and do not provide an opportunity to dissect the roles of regional DA release.

Recently introduced PD models that have a more progressive pattern of pathology have begun to provide some insight into this question. For example, our group has used intersectional genetics to selectively disrupt mitochondrial complex I function in DA neurons (González-Rodríguez et al., 2021). In this model, there is an early loss of DA signaling in the dorsolateral striatum that progresses medially and then ventrally, much like that envisioned for human PD patients (Kordower and Burke, 2018). Surprisingly, in this model, striatal DA depletion is not accompanied by parkinsonian motor deficits. Rather, mice with selective loss of striatal DA signaling have motor learning and sequencing deficits, as well as deficits in sensory-guided movement – just as one might predict from the literature described above. Levodopa-responsive parkinsonism only appears in this model when DA release in the SNr falls. This SNr signaling deficit is causally linked to parkinsonian disability, as preferentially elevating DA signaling in the SNr restores motor function. This behavioral outcome was also achieved by boosting DA signaling preferentially in the striatum, suggesting that frank parkinsonism requires a deficit in DA signaling that spans the basal ganglia. Precisely why this is the case remains to be rigorously elucidated. But one possibility is that while an imbalance between direct and indirect pathway excitability is necessary for the emergence of the disruptive synchronous, rhythmic bursting in STN and GPe, in the prodromal stages of PD, extra-striatal DA signaling blunts the propagation of this pathophysiology to other brain motor centers, enabling them to compensate for striatal dysfunction. The regional “cutoff” could be accomplished by presynaptic mechanisms (described above) that inhibit coupling of the indirect pathway to SNr/GPi neurons, while enhancing that of the direct pathway (Chuhma et al., 2011; Hadipour-Niktarash et al., 2012; Borgkvist et al., 2015; Ding et al., 2015). Thus, the loss of extra-striatal DA signaling appears to be a critical event in the onset of clinical PD.

## Moving on – a new model of basal ganglia function

Thomas Kuhn argued that science does not evolve gradually toward truth. Rather, science relies upon a paradigm which remains constant until it cannot explain a significant body of the experimental literature and is supplanted by a new paradigm that provides a unified accounting of the available data (Kuhn and Hacking, 2012). While the classical model cannot explain a large body of the experimental literature and needs to be replaced, a new paradigm remains to be developed. It might be argued that the complexity of the basal ganglia circuitry makes this an extremely difficult task. Certainly, a simple, two-dimensional representation of the detailed circuitry of the basal ganglia that is readily accessible to a broad audience (that includes students and clinicians – not just experts in the field) is becoming less tractable as more connections are revealed. Another limitation of these types of “models” is that they do not effectively capture cellular and circuit dynamics – they are static representations. For example, as noted above, basal ganglia neurons differ dramatically in their physiology – ranging from “wall-flower” SPNs that are designed to be quiescent under most circumstances to SNr neurons that are buzzing along at high spike rates in the absence of any synaptic input. These neurons should not be treated as equivalent nodes in a neural network.

One way forward is to create a more abstract, conceptual model of the basal ganglia based upon general principles of connectivity and population dynamics. It will be critical for this type of model to capture the behavior of neuronal populations in each region of the basal ganglia and the impact of the dimensionality reduction as one passes from the cortex to striatum to GPe to the SNr/GPi (Bar-Gad et al., 2003; Barack and Krakauer, 2021; Serrano-Reyes et al., 2022). In networks, like the one formed by the cortex and striatum, the computations being performed may be very difficult to reconstruct from the spiking of individual neurons (Hopfield and Tank, 1986; Beyeler et al., 2019). As a consequence, population-based models could help us see the forest for the trees. Certainly, the capacity to sample the activity of large neuronal populations in animals performing complex, “naturalistic” tasks – which is critical to this type of endeavor – has grown dramatically in recent years (Lopez-Huerta et al., 2021; Steinmetz et al., 2021). The computational tools available for this type of approach also have grown (Hjorth et al., 2020; Humphries and Gurney, 2021; Codol et al., 2022; Scott and Frank, 2022). However, it is also important not to lose sight of the fact that the computations being performed by neural networks depend upon the details and a concerted effort should be made to bridge between levels of abstraction (DePasquale et al., 2023). Why? Aside from the scientific merits of this kind of effort, a major goal of studying the basal ganglia is to develop strategies for correcting disorders that prevent it from working properly. The number of neurological disorders inextricably tied to the basal ganglia is staggering (e.g., PD, drug abuse, schizophrenia, Huntington’s disease, etc.). The interventions available to us at this point in time to correct these disorders are invariably at the molecular and cellular level. Having a model that links this level of specificity to population behavior will allow the formulation of testable hypotheses about how to fix specific disorders, something that is largely lacking in the translational effort now. Thus, there is a clear need for experimentalists and modelers to work more closely together to push this effort forward. Models are under-utilized tools by basal ganglia experimentalists and if we are to gain a better grasp of what is really going on during goal-directed action, or habit execution or internally guided exploration, this needs to change.

## Author contributions

DJS, SZ, QC, and DVS wrote and edited the manuscript. All authors contributed to the article and approved the submitted version.
